# Aluminium Phosphide-Induced Expression of Covertly Present Brugada Pattern in Electrocardiogram: A Rare Case Report

**DOI:** 10.7759/cureus.10552

**Published:** 2020-09-20

**Authors:** Satyabrata Guru, Rajesh Kumar, Anupama Behera, Subhabrata Patra, Prakash Kumar

**Affiliations:** 1 Trauma and Emergency Medicine/Internal Medicine, All India Institute of Medical Sciences, Bhubaneswar, IND; 2 Internal Medicine, All India Institute of Medical Sciences, Bhubaneswar, IND

**Keywords:** aluminum phosphide, phosphine gas, brugada pattern

## Abstract

Aluminum phosphide is a common suicidal agent in an agrarian country like India. Toxicity is mostly due to the liberation of phosphine gas, which non-competitively inhibits cytochrome oxidase in the mitochondria causing cell hypoxia. It can involve almost any organ in the body, but the most common is cardiovascular system. Various cardiovascular manifestations are hypotension, myocarditis, pericarditis, congestive heart failure, various ECG changes like myocardial infarction, conduction abnormalities, various arrhythmias, and very rarely unmasking of the Brugada pattern. Here we are presenting a case in which the patient developed unmasking of the Brugada pattern in ECG, and gradually he improved symptomatically and ECG wise with conservative treatment. As unmasking of the Brugada pattern in ECG can lead to life-threatening arrhythmias, one has to be cautious and keep this in mind while dealing with a case of aluminum phosphide poisoning.

## Introduction

Aluminum phosphide is widely used as a fumigant for stored grains to control rodents and insect pests. Because of its wide availability, it is used as a common suicidal poisoning agent. The various initial symptoms are non-specific such as nausea, vomiting, and dizziness, but within 8-10 hours patients may deteriorate and develop signs of heart failure, refractory shock, life-threatening arrhythmias, respiratory failure, and ultimately multiple organ dysfunction, leading to death. All these effects are due to cardiovascular involvement, associated with cellular hypoxia. It may affect almost any organ or system of the body because of non-competitive inhibition of cytochrome oxidase enzyme and ultimately causing energy crisis in cellular level. Gastrointestinal, renal, and neurological involvement are documented in many case reports. Mortality due to aluminum phosphide poisoning is very high, ranging from 37% to 100% [1]. Patients are usually treated symptomatically by gastric lavage with potassium permanganate, coconut oil, sodium bicarbonate solution, and vasopressors. In our case, the patient developed coved ST-segment elevation in right precordial leads with right bundle branch block (RBBB) pattern, typical of Brugada pattern in ECG, which is dangerous, because it can lead to ventricular fibrillation and death. But fortunately, in our case, the Brugada pattern was transient, mostly because of aluminum phosphide induced, as the patient improved symptomatically and ECG wise with conservative treatment.

## Case presentation

**Figure 1:**
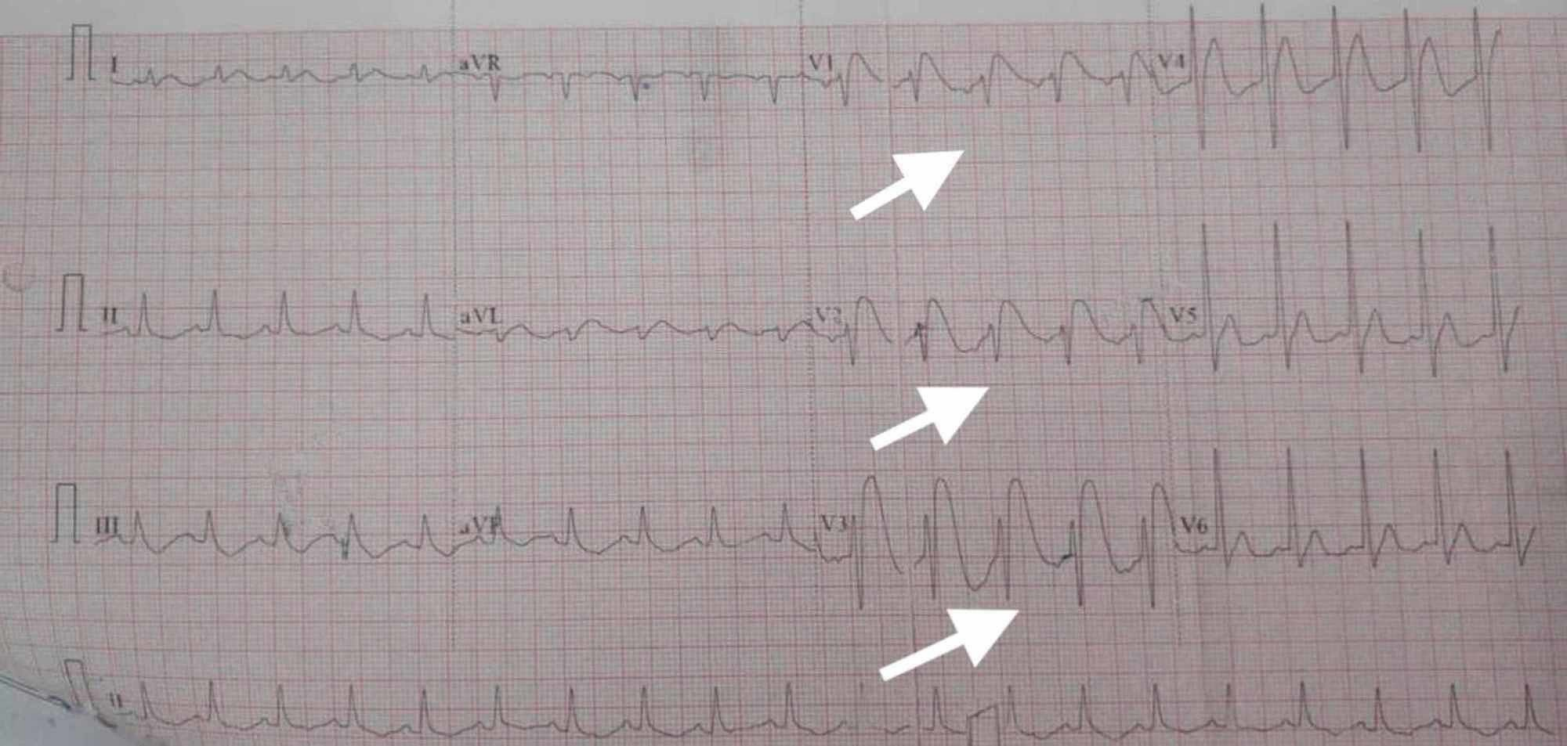
Electrocardiogram (ECG) presentation ECG showing pulse rate (PR) 119/min, corrected QT interval (QTc) 434 msec, coved ST-segment elevation of more than 5 mm in lead V1 to V3, with incomplete right bundle branch block (RBBB) morphology, and T-wave inversion in V2 and V3, suggestive of type 1 Brugada pattern.

A 19-year-old young male patient admitted to the emergency department after eight hours of consumption of aluminum phosphide poison. He was initially treated at a local hospital with potassium permanganate stomach wash, i.v. fluids, and pantoprazole injection and referred to our hospital. On arrival, his chief complaint was two episodes of vomiting, mild pain abdomen, and reeling of the head. No past history of syncope and arrhythmia was present. There was no family history of sudden cardiac death. On general examination, the patient was conscious, pulse rate (PR) 92/min, regular and low volume, blood pressure (BP) 92/48 mmHg, respiration rate (RR) 18/min, and SpO_2_ 98% on room air. Abdominal examination revealed mild epigastric tenderness, no guarding or rigidity, and bowel sounds normally heard. Other systems examination revealed no abnormalities. Arterial blood gas (ABG) was immediately done, which showed pH 7.27, pO_2_ 86.1%, pCO_2 _41, HCO_3_ 24.9 mmol/L, lactate 2 mmol/L, and normal serum electrolytes except serum magnesium, which was 1.2 mg/dL (low). Routine blood investigation showed normal liver and kidney function tests, and normal leukocyte count. Immediately ECG was advised, which revealed PR 119/min, corrected QT interval (QTc) 434 msec, coved ST-segment elevation of more than 5 mm in lead V1 to V3, with incomplete RBBB morphology, and T-wave inversion in V2 and V3, suggestive of type 1 Brugada pattern (Figure 1).

**Figure 2:**
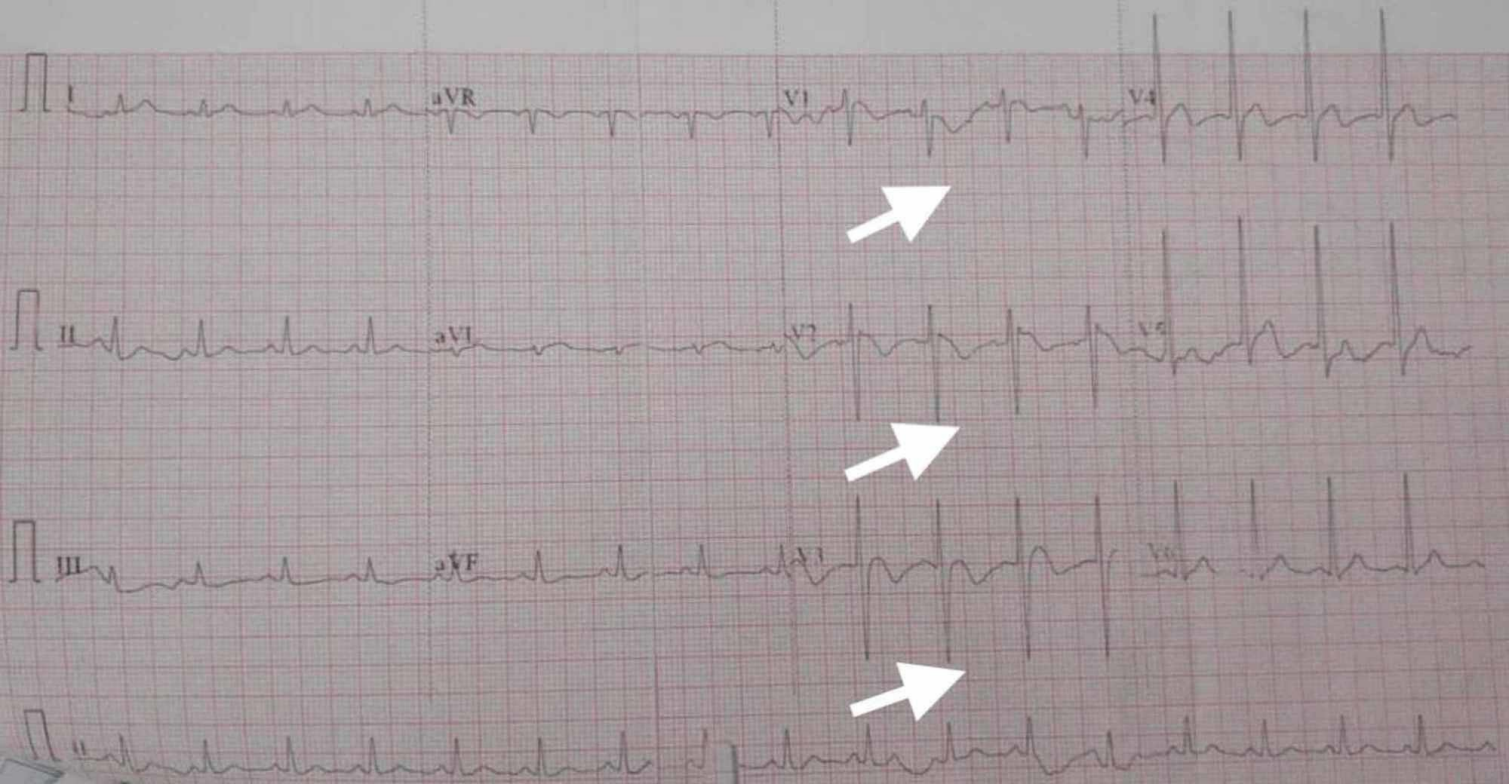
Electrocardiogram (ECG) after 12 hours of treatment ECG showing pulse rate (PR) 105/min, corrected QT interval (QTc) 398 msec, ST-segment elevation became isoelectric in lead V1 and decreased to 1 mm in lead V2-V3, and T-wave inversion in V1 to V3.

The serum troponin test was negative. The patient was managed conservatively with inj. magnesium sulfate (MgSO_4_) 2 gm i.v. infusion over 30 minutes followed by 1 gm i.v. six hourly, i.v. fluids, injection ondansetron, injection pantoprazole, and injection drotaverine. The patient was closely monitored for vitals and ECG changes. After 12 hours of treatment, the patient improved clinically. BP improved to 110/66 mmHg with only i.v. fluids. Serum magnesium becomes 2 mg/dL (normal). Vomiting, pain abdomen, and dizziness subsided with conservative treatment. ECG at 12 hours showed dramatic improvement, which showed PR 105/min, QTc 398 msec, ST-segment elevation became isoelectric in lead V1 and decreased to 1 mm in lead V2-V3, and T-wave inversion in V1 to V3 (Figure 2). The patient was finally discharged to go home after 72 hours of admission.

## Discussion

Aluminum phosphide, when ingested, liberates phosphine gas in the stomach. Phosphine is rapidly absorbed by the gastric mucosa and, once in the bloodstream, it reaches various tissues and at the cellular level inhibits the mitochondrial respiratory chain and hence leads to cell necrosis and death. Phosphine non-competitively inhibits cytochrome oxidase of mitochondria, blocking electron transport chain and oxidative phosphorylation, producing energy crisis in the cell [2,3]. Phosphine resembles cyanide in that it inhibits cytochrome oxidase and thereby hampers cellular oxygen utilization. Heart and vascular system is commonly affected, causing refractory hypotension, myocarditis, heart failure, various ECG changes like ST-segment elevation, PR prolongation, broad QRS complexes, supraventricular ectopic, atrial fibrillation, and bundle branch blocks [4]. It also causes gastrointestinal hemorrhage, renal and central nervous system toxicity, respiratory failure, and ultimately multiorgan system failure and death. Hypomagnesemia has been known to cause arrhythmias in aluminum poisoning, and magnesium supplement has been suggested as a therapeutic option [1]. In our case, the patient developed hypotension on arrival to the emergency department but was improved with i.v. fluids. He developed unmasking of Brugada syndrome (type 1) in ECG, which is not so common in aluminum phosphide poisoning. With conservative treatment with injection magnesium sulfate, i.v. fluid, injection pantoprazole, and injection ondansetron, the patient’s ECG became near normal after 12 hours and he improved significantly and discharged after 72 hours. There was no past history of syncope or arrhythmias, and no family history of sudden death. The patient's Brugada pattern (type 1), which subsequently becomes normal after 72 hours, may be categorized as an inducible Brugada ECG pattern [5]. The concealed Brugada syndrome can be unmasked by class IA and class IC antiarrhythmic drugs such as procainamide, disopyramide, and flecainide, calcium-channel blockers such as nifedipine and diltiazem, tricyclic antidepressants such as amitriptyline and desipramine, and other drugs such as lithium, cocaine, etc. Brugada syndrome is prominent right ventricular disease because of depolarizing outward current like Ito is prominent in the right ventricle and epicardium, which causes prominent action potential (AP) notch in the epicardium. The inscription of J wave is due to heterogeneous distribution of Ito and AP notch [6]. Various factors that will increase potassium current will increase ST-segment elevation, whereas causes that increases calcium current will decrease ST elevation. Magnesium acts as a cofactor of Na+ K+ ATPase that will block the cellular efflux of potassium through K+ channels in cardiomyocytes, and thereby decreases potassium current [7]. Hence, magnesium supplementation is used as a therapeutic option in case of aluminium phosphide poisoning with hypomagnesemia. In our case, the Brugada pattern was mostly induced by aluminum phosphide poisoning because of hypomagnesemia, which was present initially at the onset, and subsequently, the ECG pattern became near normal after the correction of magnesium. The fatality rate in aluminum phosphide poisoning is usually very high; the fatal dose is 0.5 gm. In our case, the patient had consumed lesser amount of aluminum phosphide, and hence survived.

## Conclusions

Various ECG abnormalities have been mentioned in the literature because of aluminum phosphide poisoning; however, induction of Brugada pattern is sparse in the literature. One has to be vigilant to look after these ECG changes, as it can precipitate into dangerous ventricular fibrillation and ultimately death. If the Brugada pattern is associated with electrolyte disturbances such as hypomagnesemia and hyperkalemia, it needs constant monitoring and urgent treatment to correct these abnormalities. As there is no specific antidote for aluminum phosphide poisoning, only symptomatic treatment and electrolyte correction is the key.
